# Synergistic Antimicrobial Activity of Ceftriaxone and *Polyalthia longifolia* Methanol (MEPL) Leaf Extract against Methicillin-Resistant *Staphylococcus aureus* and Modulation of *mecA* Gene Presence

**DOI:** 10.3390/antibiotics12030477

**Published:** 2023-02-27

**Authors:** Valiappan Ranjutha, Yeng Chen, Lamya Ahmed Al-Keridis, Mitesh Patel, Nawaf Alshammari, Mohd Adnan, Sumaira Sahreen, Subash C. B. Gopinath, Sreenivasan Sasidharan

**Affiliations:** 1 Institute for Research in Molecular Medicine (INFORMM), Universiti Sains Malaysia (USM), Gelugor 11800, Pulau Pinang, Malaysia; 2 Department of Oral & Craniofacial Sciences, Faculty of Dentistry, University of Malaya, Kuala Lumpur 50603, Selangor, Malaysia; 3 Department of Biology, College of Science, Princess Nourah bint Abdulrahman University, P.O. Box 84428, Riyadh 11671, Saudi Arabia; 4 Department of Biotechnology, Parul Institute of Applied Sciences and Centre of Research for Development, Parul University, Vadodara 391760, India; 5 Department of Biology, College of Science, University of Hail, P.O. Box 2440, Hail 81451, Saudi Arabia; 6 Faculty of Chemical Engineering & Technology, Universiti Malaysia Perlis (UniMAP), Arau 02600, Perlis, Malaysia; 7 Institute of Nano Electronic Engineering, Universiti Malaysia Perlis (UniMAP), Kangar 01000, Perlis, Malaysia; 8 Micro System Technology, Centre of Excellence (CoE), Universiti Malaysia Perlis (UniMAP), Pauh Campus, Arau 02600, Perlis, Malaysia

**Keywords:** ceftriaxone, methicillin-resistant *Staphylococcus aureus*, *Polyalthia longifolia*, synergistic effect, *mecA* gene, multiplex PCR, gene expression, skin diseases

## Abstract

Medicinal plants are an essential source of traditional curatives for numerous skin diseases. *Polyalthia longifolia* (Sonn.) Thwaites (Annonaceae family) *is a medicinal plant* used to cure skin illnesses. *P. longifolia* is usually applied in folkloric therapeutical systems to treat skin diseases. The methicillin-resistant *Staphylococcus aureus* (MRSA) bacteria is among the essential bacteria contributing to skin diseases. Hence, to verify the traditional medicinal claim of *P. longifolia* usage in skin disease treatment, the current research was performed to study the synergistic antibacterial activity of standardized *Polyalthia longifolia* methanol leaf extract (MEPL) against MRSA bacteria. The synergistic antimicrobial activity result of ceftriaxone, when mixed with MEPL, against MRSA was investigated by the disc diffusion method, broth microdilution method, checkerboard dilution test, and modulation of *mecA* gene expression by multiplex polymerase chain reaction (multiplex PCR). The MEPL extract exhibited good *synergistic* antimicrobial activity against MRSA. Using the checkerboard method, we confirmed the synergistic effect of MEPL from *P. longifolia* and ceftriaxone (2:1) for MRSA with a marked reduction of the MIC value of the ceftriaxone from 8000 µg/mL to 1000 µg/mL. Moreover, the combination of MEPL with ceftriaxone significantly (*p* < 0.05) inhibited the presence of the resistant *mecA* gene in the tested strain. The LC–ESI–MS/MS analysis identified compounds that were reported to exhibit antimicrobial activity. Conclusively, the MEPL extract, an important etiological agent for skin diseases, showed worthy synergistic antimicrobial action against MRSA bacteria, thus supporting the traditional use of *P. longifolia*.

## 1. Introduction

Microbial infectious diseases have become the third most crucial reason for mortality and morbidity worldwide. The contagion instigated by methicillin-resistant *Staphylococcus aureus* (MRSA) has contributed significantly to deadly infections and diseases [1]. There is growing proof that *S. aureus* is becoming resistant to all the standard antibiotics. Ceftriaxone belongs to a class of drugs identified as cephalosporin antibiotics and is extensively used to treat resistant bacterial strains, including *S. aureus* infection [2]. Nevertheless, disturbingly, the emergence of ceftriaxone-resistance MRSA bacteria was reported in the literature [3]. Moreover, a genetic mutation was involved in the development of resistance to the antibiotic. The attainment of the *mecA* gene by horizontal transmission by conjugation was the leading cause of antibiotic resistance in *S. aureus* [4]. This important *mecA* gene has contributed to methicillin resistance in *S. aureus* strains, which encodes a novel penicillin-binding protein 2A (PBP2A) [5]. Therefore, new alternative strategies are needed to address this issue by developing new antimicrobial agents, modifying the existing antibiotic activity with a combination of plant extracts as resistance modifying agents, or using the plant extract combined with existing antibiotics against resistant bacteria to suppress the expression of the *mecA* gene in MRSA bacteria. Consequently, the increasing incidence of MRSA bacterial infection has drawn the pharmaceutical and scientific community’s attention to studies on the potential antimicrobial activity of plant-derived substances used in traditional medicine in different countries. Scientists from divergent fields are investigating medicinal plants regarding their antimicrobial usefulness. Hence, the development of a new antibacterial against MRSA is of crucial importance.

Consequently, the search for drugs derived from medicinal plants by scientists has accelerated in recent years worldwide. The medicinal plant, a famous healthcare agent, is used daily by billions of people globally for their primary healthcare. The medicinal plant was considered a panacea with various curative values in traditional medicine, including anti-infectious activity. One crucial medicinal plant with multiple curative values is *Polyalthia longifolia* var. angustifolia Thw. (Annonaceae). *P. longifolia* is a medicinal plant with linear–lanceolate leaves found in Sri Lanka, India’s tropical parts, and Malaysia. This tree is normally planted along roadsides and gardens due to its beautiful appearance. *P. longifolia* is one of the most important traditional indigenous medicinal plants commonly used in folk medicine to treat skin diseases, fever, hypertension, helminthiasis, and diabetes [6]. The MRSA bacteria is also one of the important bacteria contributing to skin and soft tissue infection [7], which leads to major illness and death [8], comprising endocarditis, septic shock, bacteremia, and pneumonia [9]. Hence, to verify the traditional medicinal practitioner’s claims on the contribution of *P. longifolia* to skin disease treatment, the present research studied the synergistic antimicrobial action of *P. longifolia* leaf extract and ceftriaxone antibiotic against MRSA bacteria.

Until 2019, there was limited experimental evidence of the synergistic activity between *P. longifolia* leaf extract and synthetic antibiotics against MRSA. Previous experiments have demonstrated the in vitro interaction of ampicillin and *P. longifolia* leaf ethyl acetate fraction (PLEAF) by checkerboard and microscopic techniques against MRSA [10,11]. That previous study showed that the PLEAF fraction worked synergistically with ampicillin to kill MRSA’s local resistance strain. Moreover, the PLEAF fraction also exhibited excellent antioxidant activity. The combination of the PLEAF fraction with ampicillin also increased Vero cell viability. This critical finding showed the non-toxic nature of ampicillin in the presence of PLEAF in combinational therapy. Further study was also conducted to observe the in situ synergistic antimicrobial effects between PLEAF and ampicillin against a local MRSA isolate using modern scanning electron microscopy (SEM) observation [11]. PLEAF and ampicillin combination exhibited significant antibacterial activity against MRSA by killing the resistant MRSA bacteria, as observed via SEM analysis. However, as a further study in understanding multidrug-resistant bacteria’s challenges, *P. longifolia* leaf extract antibacterial activity, antibiotic modifying activity, and mutagenic effects combined with different first-line antibiotics commonly used against infectious agents should be investigated. Investigating the synergistic antimicrobial effects of the *P. longifolia* leaf methanolic extract combined with β-lactam antibiotics, such as ceftriaxone, will enhance the understanding of the synergistic antimicrobial effects of *P. longifolia* leaf extract, which has never been studied in detail before. In addition, the synergistic effect of ceftriaxone and *P. longifolia* methanol leaf extract in combination against MRSA bacteria and the *mecA* gene is still unclear, and few studies were conducted in this line. Therefore, the objective of the current research was to study the action of MEPL from *P. longifolia* on the regulation of *mecA* gene presence in the MRSA strain and study the synergistic effect of ceftriaxone and MEPL in this bacterium.

## 2. Results

### 2.1. Ceftriaxone and MEPL Antibacterial Activity against MRSA Isolates

Antimicrobial susceptibility of MRSA isolates shows complete resistance to the standard dosage strengths (8 μg/mL, 16 μg/mL, 32 μg/mL, and 64 μg/mL) of ceftriaxone, and no diameter of zone of inhibition was produced by all the different ceftriaxone dosages tested in this study (Table 1). Conversely, the tested MEPL exhibited significant antibacterial activity against MRSA by producing a clear zone of inhibition between 21 mm and 34 mm (Table 1). The negative control 5% dimethyl sulfoxide (DMSO) did not produce any zone of inhibition.

### 2.2. Determination of the MIC and MBC Concentration of Ceftriaxone and MEPL against the MRSA Isolate

The antibiotic MIC value is an essential aid in evaluating bacterial resistance. According to 2022 CLSI interpretive measures, MRSA is susceptible to ceftriaxone when the MIC value is ≤8 µg/mL, and MRSA is susceptible to ceftriaxone with a MIC value of 32 μg/mL. The MIC of ceftriaxone was obtained using the broth dilution method, and the ceftriaxone MIC value was 8000 µg/mL, visibly inhibiting MRSA growth in the broth. While the ceftriaxone MBC value, where the lowest concentration showed zero growth on sterile NA, was found at 8000 μg/mL. The MIC result demonstrated MRSA growth in a concentration of ≥62.5 μg/mL (the breaking point of ceftriaxone is ≤16 to ≥64 μg/mL). This proves that the MRSA used in this study was highly resistant towards ceftriaxone. The MEPL recorded the MIC value of 16,000 µg/mL. On the other hand, when a volume of 100 µL of inoculum from each tube was plated on fresh sterile NA, the lowest concentration of MEPL where no MRSA growth was observed was at a concentration of 16,000 μg/mL, therefore, indicating the MBC value of the MEPL to be also 16,000 μg/mL. It should be noted that the MIC and MBC results for MEPL against the MRSA strain showed a larger value than ceftriaxone.

### 2.3. Synergistic Activity of Antibiotic with MEPL

The interrelation effects between ceftriaxone and MEPL against MRSA were tested using the checkerboard technique in association with the MIC value. Ceftriaxone and MEPL combination treatment enhanced the antimicrobial effect and exhibited synergistic activity on MRSA (Table 2). In the combination treatment, the MIC values of ceftriaxone and MEPL against MRSA were reduced to eight times lower (1000 µg/mL and 2000 µg/mL). As predicted, unique antibacterial activity with a lower MIC value was demonstrated by ceftriaxone in the presence of the MEPL in the combination therapy. Coherently, it resulted in a synergistic antibacterial effect against the tested MRSA via the combination therapy of ceftriaxone and MEPL extract. 

Calculation of the FIC index of MEPL and ceftriaxone to determine the synergistic effect:

MIC of ceftriaxone alone = 8000 µg/mL
MIC of ceftriaxone in combination = 1000 µg/mL
MIC of MEPL alone = 16,000 µg/mL
MIC of MEPL in combination = 2000 µg/mL
**FIC**_ceftriaxone_ = 1000 µg/mL ÷ 8000 µg/mL = **0.125**
**FIC**_MEPL_ = 2000 µg/mL ÷ 16000 µg/mL = **0.125**

The sum of FIC (**ΣFIC**) is calculated as follows:

     **ΣFIC** = **FIC**_ceftriaxone_ + **FIC**_MEPL_
   
       = 0.125 + 0.125
   
       = **0.25**

In brief, the MIC value of the MEPL and ceftriaxone in the checkerboard test were 2000 µg/mL and 1000 µg/mL, respectively. The FIC index of the combination of MEPL and ceftriaxone was 0.25, which indicates a significant synergistic antimicrobial activity against the MRSA bacteria. The combination is considered synergistic when the ΣFIC index is ≤0.5, and indifference is indicated by an FIC index > 0.5 to ≤4, while antagonism is when the ΣFIC is >4. In addition, the initial MIC values of MEPL (16,000 µg/mL) and ceftriaxone (8000 µg/mL) were found to reduce to 2000 µg/mL for MEPL and 1000 µg/mL for ceftriaxone (*p* < 0.05), respectively, in the checkerboard test against the MRSA bacteria.

### 2.4. Presence of the mecA Gene in MRSA Treated with Different Combinations of MEPL and Ceftriaxone

#### 2.4.1. Purity of Genomic DNA

**Figure 1:**
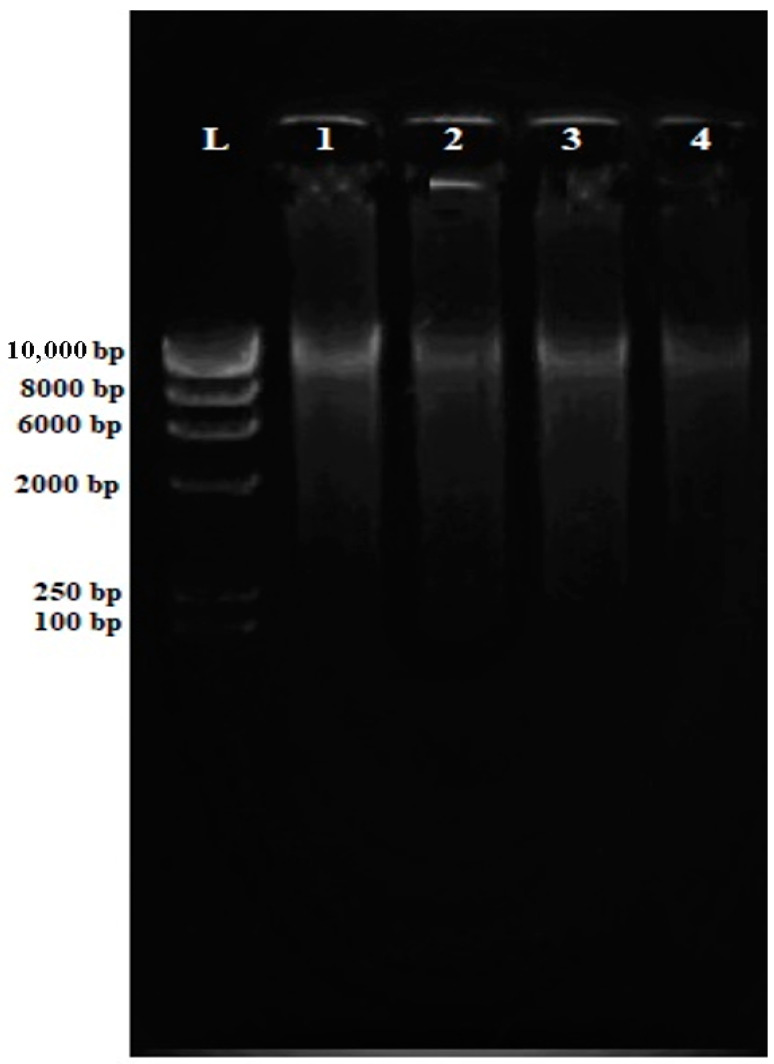
Electrophoresis gel (0.8% agarose) of the extracted genomic DNA from MRSA isolates. All genomic MRSA DNA (Lane 1–Lane 4) are intact for downstream applications.

DNA concentration, purity, and contamination are the three factors that can affect the multiplex PCR test. Nucleic acids are typically quantified (at an absorption ratio of 260 nm/280 nm) to obtain an average DNA concentration and purity necessary to be considered when carrying out PCR (Table S1). All the DNA extracted demonstrated a purity ratio value of 1.8, which indicates low protein contamination. The result was analyzed by electrophoresis on a 0.8% agarose gel followed by ethidium bromide staining to confirm an adequate amount of the DNA present with a clear band for further amplification with primary and targeted band detection. As shown in Figure 1, bands of genomic DNA can be seen on top of the gel. The absence of smearing indicates that the DNA is intact and not degraded.

#### 2.4.2. Optimization of *mecA* Gene Amplification

**Figure 2:**
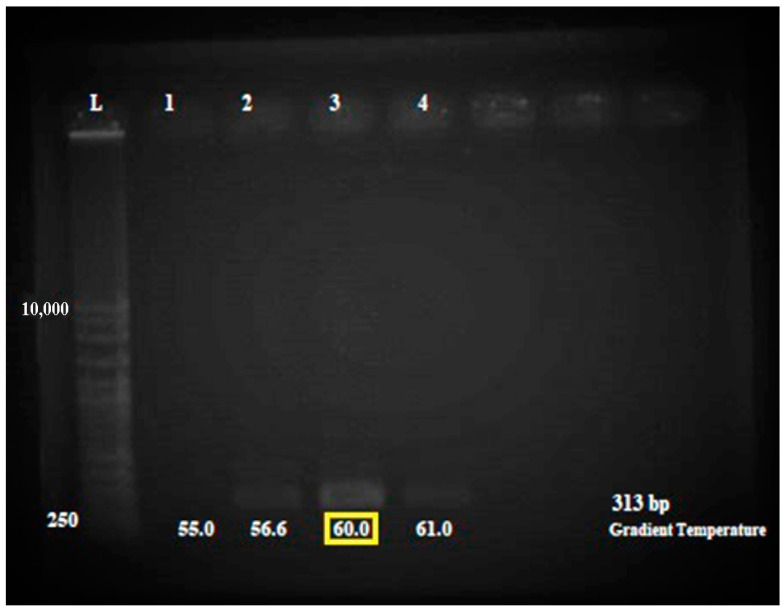
Electrophoresis gel (3% agarose) of the PCR products of the *mecA* gene for PCR optimization. The amplification optimized using MRSA isolates. Lane L = 1 kb ladder. The amplification of optimized isolate for annealing temperature 55.0 °C, 56.6 °C, 60.0 °C, and 61.0 °C. Lane 1 = amplification at annealing temperature 55.0 °C, Lane 2 = amplification at annealing temperature 56.6 °C, Lane 3 = amplification at annealing temperature 60.0 °C, and Lane 4 = amplification at annealing temperature 61.0 °C. A more apparent band was observed for the 60.0 °C reaction as shown in the yellow box.

The gradient amplification was performed to obtain the optimum annealing temperature for the multiplex PCR. Isolate 20 with a purity value of 1.7 and DNA concentration of 33.5 ng/µL (Table S1), was used throughout optimization since this isolate shows an enhanced DNA band in extracted product during electrophoresis observation. Four specific temperatures at 55.0 °C, 56.6 °C, 60.0 °C, and 61.0 °C were selected for the gradient PCR. As shown in Figure 2, a clear thick band was visible using the annealing temperature of 60.0 °C. Table S2 shows the relative intensity of the PCR amplicons on the 3% agarose gel. This finding proved that the DNA band present at 60.0 °C annealing temperature was the perfect band for *mecA* gene amplification via PCR.

#### 2.4.3. Detection of *mecA* Gene by Multiplex PCR

**Figure 3:**
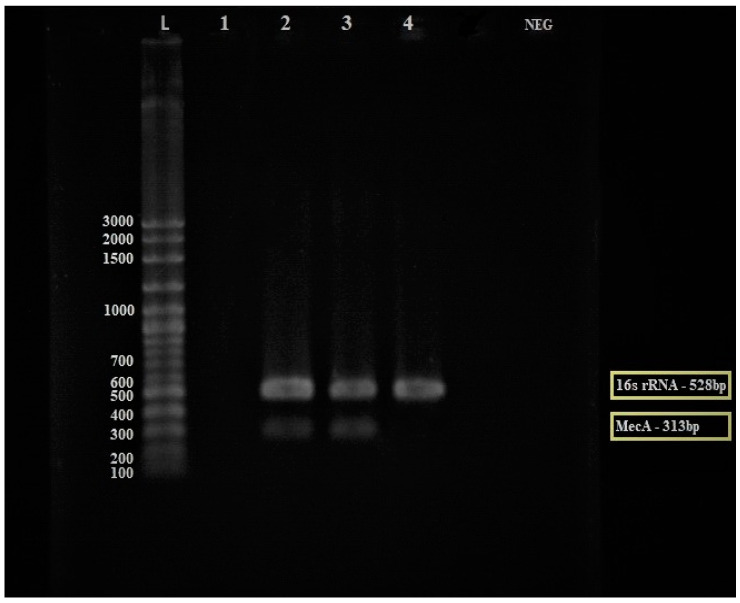
Electrophoresis gel (3% agarose) of the PCR products of the *mecA* gene for the discovery of the *mecA* gene from MRSA (Lane 2–3) and MSSA (Lane 4) isolates. Lane L = 100 bp ladder, Lane 1 = empty lane, Lane 2 and 3 = MRSA isolates, Lane 4 = MSSA control isolate, and Lane NEG = negative control. The yellow box: The *mecA* gene was expressed in MRSA strain while the *16S rRNA* gene was expressed in both MRSA and MSSA strains.

The multiplex PCR was used to detect *mecA* gene-encoded ceftriaxone resistance directly from MRSA culture using the *mecA* gene and specific *S. aureus* 16S rRNA primers as an internal control for the *16S rRNA* gene, which is a conserved region in all prokaryotic bacteria. In the MRSA bacteria, the *mecA* gene should amplify at 313 bp and the *16S rRNA* gene at 528 bp. In comparison, the methicillin-susceptible *Staphylococcus Aureus* (MSSA) should only amplify the *16S rRNA* gene at 528 bp. An MRSA confirmation test was carried out using MRSA and MSSA isolates as the control. As shown in Figure 3, the MRSA isolates successfully amplified the *mecA* gene (313 bp) and *16S rRNA* gene (528 bp), while the MSSA isolates only amplified the *16S rRNA* gene (528 bp) as predicted.

**Figure 4:**
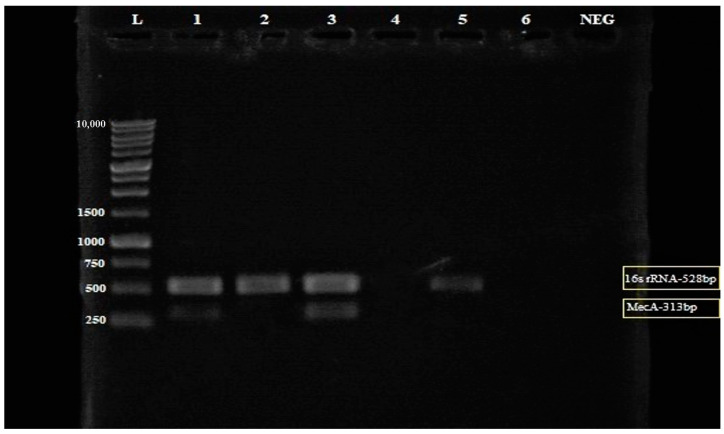
Electrophoresis gel (3% agarose) of the PCR products of the *mecA* gene and 16S rRNA for the detection of the *mecA* gene from MRSA (Lane 2–4) and MSSA (Lane 5) isolates. Lane L = 1 kb ladder, Lane 1 = Treated MRSA isolate (in a combination of 1000 µg/mL PLLME and 1000 µg/mL ceftriaxone), Lane 2 = Treated MRSA isolate (in a combination of 2000 µg/mL PLLME and 1000 µg/mL ceftriaxone), Lane 3 = untreated MRSA isolates, Lane 4 = blank, Lane 5 = MSSA isolate (control), Lane 6 = empty lane and Lane NEG = negative control. The yellow box: The *mecA* gene was not amplified in MRSA treated with the combination of 2000 µg/mL PLLME and 1000 µg/mL ceftriaxone.

Subsequently, different combinations of MEPL (1000 µg/mL and 2000 µg/mL) with ceftriaxone (1000 µg/mL) were tested against the MRSA isolate to investigate the influences of a different combinations of MEPL with ceftriaxone on the regulation of the *mecA* gene in the tested MRSA strain. As shown in Figure 4, the *mecA* gene was present in the MRSA isolate treated with MEPL and ceftriaxone at 1000 µg/mL; however, the combination of MEPL with ceftriaxone at 2000 µg/mL of MEPL and 1000 µg/mL of ceftriaxone successfully suppressed the presence of the *mecA* gene at 313 bp. In addition, as expected, the *mecA* gene was not expressed in the tested MSSA isolate.

**Figure 5:**
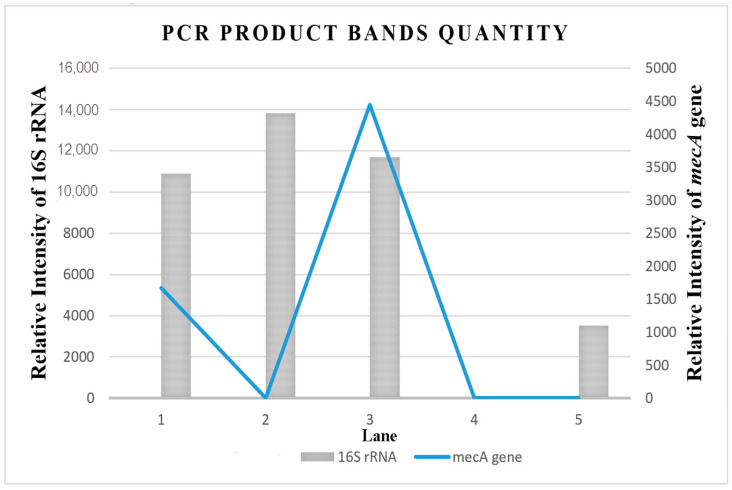
Relative band intensity by densitometry analysis of electrophoresis (3% agarose) gel of the PCR products of the *mecA* gene and 16S rRNA performed using ImageJ quantification software. Lane 1 = Treated MRSA isolate (in a combination of 1000 µg/mL MEPL and 1000 µg/mL ceftriaxone); Lane 2 = Treated MRSA isolate (in a combination of 2000 µg/mL MEPL and 1000 µg/mL ceftriaxone); Lane 3 = untreated MRSA isolates, Lane 4 = blank, Lane 5 = MSSA isolate (control).

Figure 5 shows the relative intensity (Table S3) of the amplified multiplex PCR products with the *mecA* gene and 16S rRNA bands formed on the electrophoresis gel using ImageJ software. The ImageJ software analysis of DNA bands can be used to quantify the *mecA* gene expression in the MRSA isolate. The ImageJ software analysis on the relative intensity of the *mecA* gene in MRSA provides quantitative data for the convenient evaluation of qualitative electrophoresis gel results. Therefore, with the aid of the ImageJ software, the quantification of the *mecA* gene band’s relative intensity on the electrophoresis gel was further analyzed. The finding confirmed that the combination treatment of MEPL (2000 µg/mL) with ceftriaxone (1000 µg/mL) against MRSA isolates (Figure 4, Lane 3) displays a zero value for the *mecA* gene fragment (313 bp), which indicated the complete suppression of the *mecA* gene in MRSA.

#### 2.4.4. Antimicrobial Compounds in MEPL

**Figure 6:**
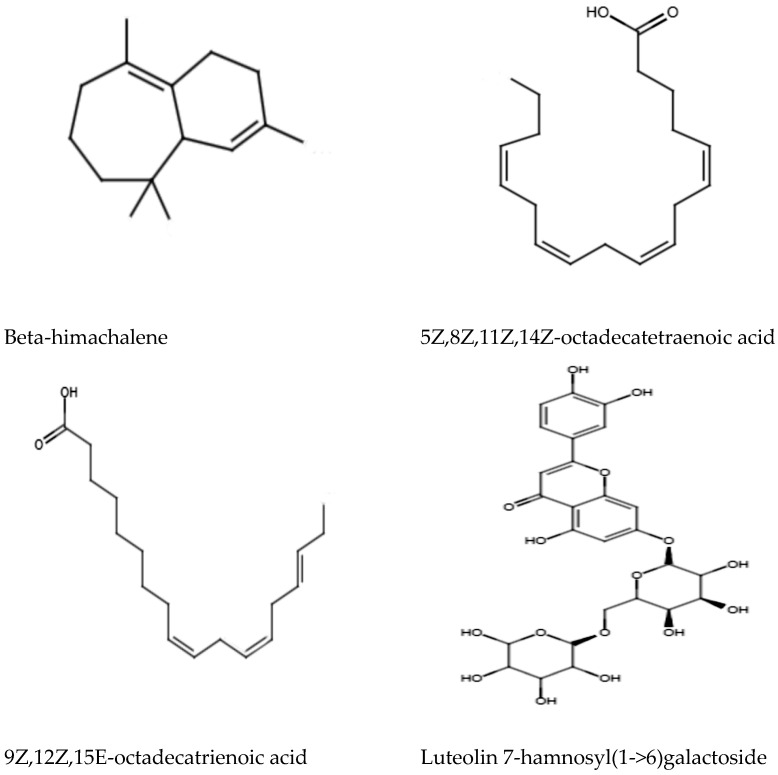
Antimicrobial phytochemical compounds found in the methanol extract of *Polyalthia longifolia* Leaf (MEPL) were detected using ultra high-performance liquid chromatography (UHPLC) equipped with the chemical library.

Ultra High-Performance Liquid Chromatography (UHPLC) analysis was performed to analyze and tentatively annotate the extracted metabolites in the MEPL with the aid of the chemical library of Metlin_AM_PCDL-N-170502.cdb. The UHPLC analysis of the MEPL showed the presence of several antimicrobial phytochemicals. Among these, beta-himachalene (1.9%), 5Z,8Z,11Z,14Z-octadecatetraenoic acid (8.3%), 9Z,12Z,15E-octadecatrienoicc acid (6.1%), and luteolin 7-rhamnosyl(1->6)galactoside (5.7%) were the antimicrobial compounds in MEPL extract. The chemical structures of the antimicrobial phytochemical compounds found in MEPL are presented in Figure 6.

## 3. Discussion

Methicillin-resistant *Staphylococcus aureus* (MRSA) infection has become one of the most historic pathogenic bacterial infection associated with health issues in developing countries. Moreover, the crucial *mecA* gene contributes to methicillin resistance in MRSA strains, which encodes a novel penicillin-binding protein PBP2a. The global trend has represented a rise in MRSA infections with the high emergence of multidrug-resistant strains [12]. This bacterium has shown resistance to various antibiotics such as methicillin, penicillin, and amoxicillin, including ceftriaxone. Ceftriaxone is a third-generation cephalosporin and remains one of the most commonly used antibiotics for antimicrobial therapy due to its efficacy and low therapeutic index [13,14]. It is reported that ceftriaxone has a broad potency spectrum against Gram-positive and Gram-negative bacteria [15]. In addition, ceftriaxone is used frequently to treat MSSA infections [16,17]. It has been used as a first-line treatment against bacteremia alongside other antibiotic combinations [18]. The rise of microorganism resistance towards third-generation cephalosporins is a global burden and has led to antimicrobial treatment failure. The bacterial organism becomes inherently resistant to the increased use of antibiotics at a higher antibiotic dosage [19,20]. Besides, bacterial resistance to an antibiotic can also be attributed to random genetic mutation [21] or the uptake of plasmid DNA (horizontal gene transfer) from foreign cells [22]. Hence, MRSA has become the center of this public health concern due to its high virulence and resistance to a broad spectrum of antibiotics [23]. This widespread organism causes challenges to both the healthcare system and patients due to increased hospitalization costs and notable mortality/morbidity rates [24]. In addition to this complication, antibiotics often produce adverse effects, namely hypersensitivity, immune suppression, and allergic reactions [25,26,27,28]. The need to develop new antimicrobials as an alternative to synthetic antibiotics for MRSA treatment is achieved from various sources. Many developing countries commonly use medicinal plants in the treatment of multiple health complications. The application of medicinal plant extracts rich with pharmacological activity, such as *P. longifolia* and its associated phytochemicals, can significantly contribute to the treatment of infectious diseases. Hence, the current research was performed to evaluate the synergistic antibacterial activity of natural MEPL in combination with ceftriaxone against MRSA bacteria.

The synergistic antimicrobial action between medicinal plant extracts and conventional antibiotics has been extensively studied to overcome the antibiotic resistance problem [29,30,31]. Synergism takes place when two different molecules interact and strengthen their actions. On the other hand, any reduction in activity from the combination treatment is termed antagonism [32]. The synergistic properties of MEPL with ceftriaxone against MRSA were evaluated in this study. The results indicated positive synergism in the combination treatment of MEPL and ceftriaxone compared to ceftriaxone or MEPL alone against the MRSA bacteria. The MIC and MBC values of ceftriaxone and MEPL decreased in the combination treatment, indicating the synergistic antimicrobial activity of MEPL in combination with ceftriaxone. MEPL may promote synergistic antimicrobial properties by acting as synergistic activity enhancers in combination with ceftriaxone, enhancing the overall antibiotic effect. The advantages associated with the synergistic interactions are that synergism effect increases treatment efficiency, decreases undesirable side effects of the single drug, such as diarrhea, nausea, bloating, and indigestion, increases the bioavailability of free agents, and an adequate therapeutic effect is achieved with comparatively smaller doses when compared with individual synthetic antimicrobials [33]. Many researchers have reported that combination therapy, mainly plant extracts with synthetic antibiotics, exhibited a synergistic effect against *S. aureus* [34,35,36,37]. Interestingly, a recent study reported impaired cell division, extensive wrinkles, cell shrinkage, and the emergence of rougher cells with fibrous matrix and clustered cells, highlighting the synergistic effect of ethyl acetate *P. longifolia* in combination with ampicillin against MRSA cells [10,11]. Another study has also suggested that the membrane-disrupting activity of combination therapy between Trp-containing antimicrobial peptides (AMPs) with four classes of traditional chemical antibiotics, namely penicillin, ampicillin, and erythromycin, increases the access of small molecule antibiotics to the cell, which allows the synergistic activity to improve antimicrobial agents’ effectiveness, increasing bacterial killing and prevent resistance development [38]. Moreover, AL-Ali et al. [39] reported the synergistic antimicrobial activity of various plant extracts in combination treatment against multi-drug resistance (MRSA) *S. aureus*. The combination of four plant extracts, namely *Mentha cervina*, *Mentha longifolia*, *Ocimum basilicum,* and *Origanum vulgare* showed good synergistic antibacterial activity against the multi-drug resistance (MDR) *S. aureus*. Besides, another independent study has reported the antimicrobial activities of the methanol, acetone, and 1,4-dioxan fractions of *P. longifolia* leaves [40]. The tested sample showed better antibacterial activity against Gram-positive bacterial and fungal strains than the Gram-negative bacterial strains studied.

Various secondary metabolites in the MEPL, as reported in the literature, such as flavonoids, alkaloids, and diterpenoids [41], can be responsible for the observed antimicrobial properties of the MEPL. Hence, screening of MEPL was performed to annotate the chemical profiles using UHPLC analysis equipped with the chemical library of Metlin_AM_PCDL-N-170502.cdb to identify the bioactive chemical constituents that could be responsible for the observed antimicrobial activity. UHPLC analysis led to the detection of the various chemical constituents, as shown in Figure 6. Moreover, the presence of himachalene and its derivatives [42], fatty acid octadecatetraenoic (9Z,12Z,15E-octadecatrienoicc acid and 5Z,8Z,11Z,14Z-octadecatetraenoic acid) [43,44], and luteolin and its derivatives [45] compounds were found in MEPL, which were previously reported to show good antimicrobial activity against various microbes including *S. aureus*, which might have contributed to the observed antimicrobial activity of the MEPL in this study. Besides, rutin was used to standardize the MEPL extract in this study since rutin enhanced the antibacterial activities, as reported in the literature [46]. As observed in this study, rutin also might contribute to the synergistic effect of the MEPL extract.

In addition, various reports in the literature reported the isolation of compounds from *P. longifolia* with antimicrobial and synergistic antibacterial activity. Interestingly, seven antimicrobial clerodane diterpenoids, namely 16(R and S)-hydroxy-cleroda-3,13(14)Z-dien-15,16-olide, 16-oxo-cleroda-3,13(14)E-dien-15-oic acid, methyl-16-oxo-cleroda-3,13(14)E-dien-15-oate, 2-oxokolavenic acid, 16(R and S) hydroxy-cleroda-3,13(14)Z-dien-15,16-olide-2-one, (4→2)abeo-16(R and S)-hydroxy-cleroda-2, 13(14)Z-dien-15, 16-olide-3-al, and 3β,16α-dihydroxy-cleroda-4(18), 13(14)Z-dien-15,16-olide [47] were isolated from the methanol extract of *P. longifolia* leaves, which are widely reported for their antibacterial and antifungal properties [48]. Furthermore, diterpenoids induce bacterial membrane disruption [49], which may allow other compounds to enter cells to initiate antibacterial activity in a combination therapy mode. Therefore, the presence of diterpenoids [49] and flavonoids [50] in the MEPL, as reported in the literature, can be hypothesized to be synergistic and enhance the antibiotic function by disrupting the membrane of the MRSA and making it susceptible to ceftriaxone. In particular, the presence of clerodane diterpene 16α-hydroxycleroda-3, 13 (14) Z-dien-15, 16-olide (CD) has been reported to be synergistic against MRSA through the disruption of the cell membrane [51]. In addition, the combination of CD, a bioactive compound in MEPL, reduced the MIC of fluoroquinolones, such as norfloxacin, ciprofloxacin, and ofloxacin, against MRSA through significant inhibition of the efflux pump [52]. Efflux pumps have been cited as the main reason for the emergence of multidrug resistance bacteria towards various antibiotics among Gram-positive and Gram-negative bacteria [53]. It was reported that CD downregulates the expression of efflux pump genes, such as *norA*, *norB*, *norC*, *mdeA,* and *mepA,* which are the genes responsible for expelling antibiotics outside the *S. aureus* cells [54]. Therefore, it can be deduced that the bioactive compounds in the MEPL may play a similar role in inhibiting the efflux pump in *S. aureus* and synergistically reversing the resistance of MRSA towards ceftriaxone.

This study also attempted to assess whether the combination of MEPL with ceftriaxone influences the presence of the *mecA* gene by observing the presence of the *mecA gene* on the agarose gel upon treatment. In this study, MEPL from *P. longifolia* with ceftriaxone inhibits the manifestation of the resistant *mecA* gene in the studied strain. In the presence of β-lactam derivatives, the MRSA strains will not demonstrate growth inhibition and can retain their capacity to expand the zone of inhibition [55]. The methicillin-resistant *mecA* gene in MRSA isolates encodes PBP2a, a transpeptidase that inhibits the antibiotic’s antimicrobial action. Another study has reported that the *mecA* gene can be a useful molecular marker for MRSA isolates [56]. In contrast, *S. aureus* isolates lacking *the mecA* gene can be considered as MSSA strains [57]. The *mecA*-positive strains differ in the expression levels to methicillin resistance, which may be complex and difficult to diagnose [58]. Therefore, molecular techniques, such as polymerase chain reaction (PCR), are suitable for detecting the methicillin resistance *mecA* gene. The multiplex PCR technique utilized in this study is a rapid tool and considered the “gold standard” for detecting the methicillin resistance *mecA* gene due to its efficacy and accuracy [59]. Optimization of the PCR protocol is routine and necessary for better sensitivity and specificity. Adequate DNA templates and optimum annealing temperature are crucial factors for successfully amplifying the *mecA* gene [60]. This was evidently supported by the current research results, where the positive control MRSA isolate amplification was improved with the appropriate annealing temperature and DNA template.

Besides, the influence of MEPL on the *mecA* gene in MRSA bacteria was proven by the finding of the checkerboard method conducted in this research to assess the synergistic action of MEPL and ceftriaxone. The checkerboard method results indicate the synergistic effect of ceftriaxone combined with MEPL against MRSA by enhancing the antimicrobial effect. The *mecA* gene analysis in the MRSA treated with ceftriaxone (1000 µg/mL) combined with MEPL (2000 µg/mL) by multiplex PCR examination showed the absence of the *mecA* gene band. This finding indicated that the gene-specific primers could not identify and bind to the region coding the *mecA* gene. This finding disclosed the effective influence of MEPL on inhibiting the presence of the *mecA* gene in MRSA bacteria. The combination of ceftriaxone and MEPL influenced the presence of the *mecA* gene in MRSA to make the local strain susceptible to ceftriaxone. Interestingly, several studies report on the influence of medicinal plant extracts on bacterial gene expression, namely *T. integrifolia, Eurycoma longifolia* Jack, and *Helmintostachys zeylanica* against *Salmonella typhimurium* strains via the Ames Test [56,61]. Alkaloids, such as β-carboline, have been a vital influence against bacterial DNA [57,58,62,63]. It was reported that β-carboline alkaloids, such as harman and harmine of *Passiflora* spp. (Passifloraceae), are responsible for DNA damage of *Saccharomyces cerevisiae* [59,64]. In another study, the mutagenic properties of the methanolic extract of *Byrsonima crassa* Niedenzu was reported due to amentoflavone. Plant extracts containing flavonoids, such as Quercitin, have also been implicated in mutagenesis [60,65]. Therefore, flavonoids [50] and alkaloids [61,66] in MEPL might be responsible for the observed suppression of the expression of the *mecA* gene, which warrants further detailed studies.

The present research studies the antimicrobial effects and modulation of *mecA* gene expression by MEPL combined with ceftriaxone against an MRSA strain for possible application as a natural product agent. The MEPL combination with ceftriaxone exhibited vigorous antimicrobial activity against the MRSA isolate. Moreover, MEPL showed a synergistic antibacterial effect with ceftriaxone against the tested MRSA strain and suppressed the presence of the resistant *mecA* gene. From the findings of this research, it was established that MEPL could reinstate the effectiveness of ceftriaxone against MRSA. Consequently, the findings of this research propose that the MEPL and ceftriaxone combination could develop novel natural remedies based on combination antibiotics therapy against MRSA infection. Furthermore, various in vitro and in vivo experiments, such as the genotoxic effect evaluated via plasmid relaxation assay, acute oral toxicity studies in animal models, and the *Allium cepa* assay [67], showed that MEPL was not toxic and safe in human applications. The in vivo acute oral toxicity study showed that MEPL was safe even at a single dose of 5000 mg/kg body weight in female albino Wistar rats. Besides, the literature also reports that MEPL exhibits various biologically beneficial effects. The antimicrobial activity of *P. longifolia* leaf extracts were also reported by Chanda and Nair [40] against 91 clinically significant pathogenic microbial strains. The polyphenol-rich MEPL exhibited good antioxidant and hepatoprotective activities against paracetamol-induced oxidative damage [67,68]. Besides, the MEPL also supported the X-ray irradiated mouse survival rate increases compared to 100% mortality in the untreated mice [69], and renoprotection against radiation-induced nephropathy by an anti-oxidative molecular mechanism [70]. These findings highlight that MEPL decreased oxidative stress and nephropathy in rats due to its anti-inflammatory activities. Moreover, MEPL also showed good cytotoxicity against HeLa cancer cells via inducing apoptotic cell death and miRNA regulation [71,72,73,74]. A recent study also showed that MEPL exhibited good antiaging activities in S. cerevisiae by modulating oxidative stress, enhancing GSH content, and increasing SOD and SIRT1 gene expression [75].

## 4. Materials and Methods

### 4.1. Polyalthia longifolia Leaf

Mature leaves of *P. longifolia* were collected (Voucher specimen number: USM/HERBARIUM/11306) from University Sains Malaysia (USM), Pulau Pinang, Malaysia main campus, in February 2020. The leaves were rinsed thoroughly with tap water and air dried under shade inside the laboratory for about 2 weeks until the leaves were dried entirely. Dried leaf parts were homogenized to a fine powder using a regular blender and stored in airtight bottles.

### 4.2. Preparation of Polyalthia longifolia Leaf Extract

Dried powder *P. longifolia* leaves then underwent methanol extraction using a cold percolation process on a rotary shaker [76]. A mass of 100 g of dried powder of *P. longifolia* leaves was added into a conical flask and soaked in 1000 mL of methanol. The flask was sealed with aluminum foil and kept on a rotary shaker (Ohaus, Parsippany, NJ, USA) at 190–220 rpm for 3 days. After 3 days, the content of the flask was filtered at different levels, initiated via 8 layers of muslin cloth followed by Whatman No. 1 filter paper to get the crude extract. The filtrates were then collected and concentrated in a rotary vacuum evaporator (Eyela, Bohemia, NY, USA) [77] at 120 rpm and 200 pi at 41 °C overnight to remove solvents from samples through the evaporation process. The concentrated extract was then collected in a glass Petri dish, kept in the oven (60 °C), and incubated to remove excessive methanol further from the sample. A constant weight of the completely solvent-free filtrates was obtained after incubation in the oven. The filtrates were then stored at 4 °C in air-tight bottles. The final product of the methanol extract of *Polyalthia longifolia* leaf (MEPL) was used to conduct the antibacterial study. The MEPL stock solution was dissolved and prepared in 5% DMSO at a final 10 mg/mL concentration. The rutin measure in MEPL extract was established on the peak area calculated from the calibration curve equation of commercially (Sigma-Aldrich, St. Louis, MO, USA) available rutin (5, 10, 100, 400, 600, 800, and 1000 µg/mL) compound (standard) (*y* = 275,885x, *r*^2^ = 0.9977). The amount of rutin in the MEPL was found to be 8.96 µg (0.896%) in 1000 µg [69].

### 4.3. Test Microorganism Collection and Maintenance

The Gram-positive bacterium MRSA and MSSA were collected from the Penang General Hospital Microbiology Unit (GH), Penang, Malaysia. The MRSA and MSSA strains were aseptically removed with an inoculating loop and streaked in a zig-zag pattern onto the freshly prepared nutrient agar (NA) plate. The MRSA and MSSA strains on the NA plate were grown for 24 h at 37 °C. The stock culture was then stored at 4 °C. The stock culture was sub-cultured every 3 weeks to maintain viability.

#### Antimicrobial Susceptibility Test

Antimicrobial susceptibility testing (AST) of bacterial isolates is a collective and significant technique in most clinical laboratories. In this study, AST was conducted using the Kirby Bauer technique [78] based on the Clinical Laboratory Standard Institutions [79] guidelines on molten Mueller Hinton Agar (MHA). The steps involved in this assay are as follows.

### 4.4. Culture Media and Inoculum Preparation

The test organisms were grown on molten Mueller Hinton Agar (MHA) at 37 °C during the antibacterial susceptibility test. The molten MHA was prepared according to the manufacturer’s instruction (Oxoid, Basingstoke, UK), autoclaved and poured onto sterile Petri dishes, and solidified at room temperature. An inoculum suspension (1.5 × 10^8^ cells/mL) equal to 0.5 McFarland was prepared by inoculating 5 similar colonies with a wire loop in up to 5 mL of tryptone soya broth (TSB) and incubated at 37 °C for 8 h up until mild-to-moderate turbidity growths could be seen.

### 4.5. Agar Disc Diffusion Assay of Ceftriaxone

A sterile cotton swab was dipped into the prepared inoculum of the MRSA suspension, which was rotated resolutely against the tube’s upper inside wall to rapidly removed excess fluid and then streaked through the entire surface of MHA plates. The plate was allowed to dry at room temperature with the inoculum, with the lid in place, for about 10 min. Standard antibiotic discs of ceftriaxone (8 μg/mL, 16 μg/mL, 32 μg/mL, and 64 μg/mL), also known as blank cartridges (Oxoid, Basingstoke, UK), were placed on the upper layer of the seeded agar plate and gently pressed on the disc’s handle while making sure all the discs were completely attached to the medium. The plates were incubated for 24 h at 37 °C. The formation of a clear zone of inhibition of ≥21 mm in diameter was considered a significant susceptibility of the organism to the MEPL extract. The experiment was replicated three times, and the mean value is displayed in this study. By measuring the diameter of the zone of inhibition, the antimicrobial activity was determined and recorded in millimeters with the aid of sliding calipers, and the organisms present were classified as sensitive, resistant, or intermediate, referring to CLSI guidelines (Table 4). The 5% DMSO was used as a negative control.

### 4.6. Agar Well Diffusion Method of Antibacterial Susceptibility Test for MEPL

The agar well diffusion method evaluated the antimicrobial activity of the MEPL with certain modifications. MRSA was grown on nutrient broth (NB) and incubated at 37 °C for 24 h. A total of 1600 μL of overnight NB culture was added to 120 mL of molten MHA and mixed well; the mixture was then poured into a sterile Petri dish and set aside to allow the plate to dry at room temperature. A sterile 5 mm in diameter cork-borer was used on the set agar to create wells. Subsequently, 25 μL of diluted plant extract in a sequence of 8 mg/mL, 7 mg/mL, 6 mg/mL, 5 mg/mL, 4 mg/mL, 3 mg/mL, 2 mg/mL, and 1 mg/mL was applied to the wells, and the plates were then incubated at 37 °C overnight. The bacterial growth was assessed based on the diameter of the inhibition zone. The tests were performed in triplicate, and average values were recorded. A 5% DMSO solution was used as a negative control.

### 4.7. Evaluation of the Minimum Inhibitory Concentration (MIC) and Minimum Bactericidal Concentration (MBC) of the MRSA Isolate against Ceftriaxone and MEPL

#### 4.7.1. Determination of the MIC of Ceftriaxone and MEPL against the MRSA Isolate

To determine the MIC concentration of ceftriaxone and MEPL against the MRSA isolate, the broth dilution method was used under the CLSI guideline. Two-fold serial dilutions of the ceftriaxone at a concentration of (8000—62.5 μg/mL) and the MEPL in the arrangement of 16,000–62.5 μg/mL in 5% DMSO were prepared in sterile capped universal bottles. Subsequently, 2 mL of overnight incubated (37 °C) MRSA suspension was added to 2 mL of each concentration of the antibiotic ceftriaxone and MEPL dilution followed by vortexing and 18 h incubation at 37 °C. The negative control comprised MHB and ceftriaxone antibiotic, while the positive control was MHB and MRSA suspension. Another 2 mL broth prepared in the universal bottle was inoculated MRSA and kept overnight in a refrigerator at 4 °C separately. The tube was used as a standard for determining complete inhibition. The MIC value was determined as the lowest concentration of ceftriaxone and MEPL inhibiting the MRSA by referring to turbidity. Besides, comparing to the standard tube incubated previously in the refrigerator was used to assess the inhibition of the organism’s growth.

#### 4.7.2. Determination of the MBC of Ceftriaxone and MEPL against the MRSA Isolate

Immediately after the MIC determination, the tubes with ceftriaxone and MEPL inhibiting the MRSA growth were used to determine the MBC. Subsequently, about 100 µL of the inoculum was added to a sterile NA media plate and incubated for 24 h in a 37 °C incubator to observe possible bacterial growth. The lowest concentration of ceftriaxone and MEPL in the subculture that showed no bacterial growth on the plate was considered the MBC [80].

### 4.8. Investigation of the Synergistic Properties of MEPL with Ceftriaxone

#### 4.8.1. Preparation of MEPL and Ceftriaxone for Synergistic Study

Two-fold serial dilutions of the extracts (16,000–62.5 µg/mL) and ceftriaxone (8000–62.5 µg/mL) were prepared. A combination drug was prepared at a ratio of 1:1 of MEPL:ceftriaxone from the highest to lowest concentration to investigate of the synergistic properties of MEPL with Ceftriaxone.

#### 4.8.2. Measurement of the Fractional Inhibitory Concentration (FIC) by Checkerboard Analysis

Ninety-six well microtiter plates were used to measure the FIC concentration for synergistic activity between MEPL and ceftriaxone [81,82]. The inoculum suspension was prepared in MHB. A total volume of 100 µL of two-fold dilution of EPL/ceftriaxone combination (1:1 ratio) was added to 900 µL of the inoculum suspension into each well of the microtiter plates, bringing the final total volume to 1 mL. The ceftriaxone was placed in columns in ascending concentrations starting at zero MIC and ending at two times the MIC. The MEPLs were similarly distributed among the rows. Accordingly, each well of the 96-well microtiter plate had a unique combination of different concentrations of the antibiotic and MEPL. Two control wells were preserved for each test batch. These included the test control (the well containing MEPL/antibiotic and the medium without inoculum) and organism control (the well containing the growth medium and the inoculum). The plate was incubated overnight at 37 °C. The MIC value was determined as the lowest concentration of ceftriaxone and MEPL inhibiting the MRSA by referring to turbidity.

##### Calculation of the Fractional Inhibitory Concentration (FIC) Index

The ΣFICs were computed with the formulae below [83]:

- FIC of plant extracts = MIC of MEPL in combination/MIC of MEPL alone
- FIC of antibiotic = MIC of antibiotic in combination/MIC of antibiotic alone

FIC index = FIC of MEPL + FIC of antibiotic

Synergy was defined as a FIC index ≤ 0.5.

The additive effect was defined as a FIC index > 0.5 but ≤4.0.

Antagonism was defined as a FIC index > 4.0.

### 4.9. Presence of the mecA Gene in MRSA Treated with Different Combinations of MEPL and Ceftriaxone

#### 4.9.1. Concentration-Dependent Assay of Ceftriaxone and MEPL against MRSA and MSSA Isolates

The one-day-old cultures of MRSA and MSSA isolates were inoculated in 50 mL MH broth and incubated at 37 °C at 120 rpm agitation. The next day, the MRSA and MSSA isolates were treated with different combinations of plant extract dosages and antibiotics as 1000 μg/mL ceftriaxone with 1000 μg/mL or 2000 μg/mL MEPL. The cultures were incubated at 37 °C at a 120 rpm agitation rate for 24 h. The next day, the culture was pelleted at 0.12× *g* (120 rpm) speed for 10 min in a tabletop centrifuge. The pellets were then subjected to genomic DNA extraction.

#### 4.9.2. Genomic DNA Extraction

The ready-to-use DNA mini kit from Stratec Molecular GmbH Berlin, Germany, was used to separate bacterial DNA from MRSA and MSSA strains. The genomic DNA of MRSA and MSSA strains was purified using the bacterial DNA purification mini kit (Stratec Molecular, Berlin, Germany) following the manufacturer’s protocol, and stored at −20 °C.

#### 4.9.3. DNA Quantification

The eluted genomic DNA was quantified by measuring UV absorption using a NanoDrop spectrophotometer (BioRad, Hercules, CA, USA). The integrity of each eluted DNA sample was evaluated by subjecting it to 0.8% (*w*/*v*) agarose gel electrophoresis analysis, and the DNA samples were kept at −20 °C for future analysis.

#### 4.9.4. Multiplex Polymerase Chain Reaction (PCR)

The genomic DNA was further subjected to multiplex PCR amplification to detect targeted genes (*mecA* and *16S rRNA*). The multiplex PCR amplification was carried out using a Bio-Rad thermal cycler. PCR was carried out in 50 μL reaction mixtures, 25 μL Quick-Load 2X power Taq Master Mix applied with 1 μL reverse primer (1 µM) and 1 μL (1 µM) forward primer and genomic DNA (30 ng/µL). Sterile distilled water was added to bring the total volume to 50 μL. The negative control comprised just the Quick-Load 2X power Taq Master Mix, primers, and sterile water. The list of primers used in this study is listed in Table 5. The conditions of the gradient multiplex PCR (30 cycles) used in this study are as follows: denaturation at 95 °C for 30 s, annealing at 55–61 °C for 30 s, and eventually elongation at 72 °C for 30 s [84]. After the optimization of the annealing temperature, a conventional multiplex PCR was carried out using the same conditions of 95 °C for 30 s, followed by annealing at 60 °C for 30 s, and eventually elongation at 72 °C for 30 s. All PCR products were then assessed using 3% (*w*/*v*) gel electrophoresis.

#### 4.9.5. Agarose Gel Electrophoresis Analysis of the PCR Product

The agarose gel (3%, *w*/*v*) was prepared using 3 g gel powder dissolved in 100 mL of TBE buffer before microwave heating for up for 2 min. A 1 μL loading dye ratio to 3 μL PCR liquid (1:3) was used for all reactions. A volume of 4 μL of the sample and appropriate DNA ladder was loaded into each well and ran at a constant 65 V power supply for 40 min. Once the bromophenol blue stain hit more than two-thirds, the gel was examined by staining with ethidium bromide under a UV trans-illuminator (Appleton Woods, Birmingham, UK) to observe the specific band locations of the amplified DNA, which were recorded in an automatic gel documentation scanner. The intensity of the bands was quantified by using the ImageJ software.

### 4.10. LC–ESI–MS/MS Identification of Antimicrobial Compounds in MEPL

Identification of the antimicrobial compounds was carried out by using the Agilent 1200 series Ultra High-Performance Liquid Chromatography (UHPLC) system (Agilent Technologies, Santa Clara, CA, USA), coupled with an Agilent 6520 Accurate-Mass quadrupole-time of flight (Q-TOF) mass spectrometer with a dual electrospray ionization source (ESI). The UHPLC system, equipped with the chemical library of Metlin_AM_PCDL-N-170502.cdb, consisted of a vacuum solvent degassing unit, a capillary pump, and an automatic sample injector. The ESI operated in positive and negative modes with an m/z range from 100–3200. ESI conditions were as follows: fragmentor voltage 125 V; nebulizer pressure 45 psi; capillary voltage 3500 V; gas temperature 300 °C, gas flow 10 L/min, and skimmer 65 V. The chromatography was performed using Agilent Zorbax Eclipse XDB-C18, Narrow-Bore 2.1 × 150 mm, 3.5 microns (Agilent Technologies, Santa Clara, CA, USA). The auto-sampler compartment was maintained at 4 °C, and the mobile phase was 0.1% formic acid in water (A) and 0.1% formic acid in acetonitrile (B). The multi-step linear gradient was applied as follows: 5% solvent B for 5 min and the gradient keep isocratic at 100% solvent B from 20 min to 25 min. The initial condition was held for 5 min before the subsequent analysis. The injection volume was 1 μL, and the flow rate was 0.5 mL/min. The chromatographic separation was performed using C_18_ column (Agilent Eclipse XDB-C18 Narrow-bore, 150 mm × 2.1 mm, 3.5-micron) and the column temperature was 25 °C. The compounds in MEPL were identified via the Metlin database by using the spectra of chromatograms obtain from liquid chromatography mass spectrometric analysis, which determined the molecular mass of the compounds in the crude extract. The mass spectra of the compounds derived from UHPLC were run against the Metlin_AM_PCDL-N-170502.cdb library for the identification of homologous compounds via Agilent Mass Hunter software. The determination of the novelty of the identified compounds was performed on Scifinder software. Conversely, previously testified compounds were subjected to a literature search for biological activities, specifically for antimicrobial activity.

## 5. Conclusions

In conclusion, the results obtained in this study demonstrate the potential of MEPL to be a candidate for combination therapy against MRSA bacteria because it has a synergistic antibacterial effect with ceftriaxone in the tested strain. The killing effect of the combinatorial treatment is connected with the inhibition of the presence of the *mecA* gene in staphylococcal resistance to β-lactams antibiotics. The antimicrobial compound analysis the in MEPL extract showed the presence of several antimicrobial compounds known for their antibacterial activity. These discoveries provided a novel choice for clinicians to use natural MEPL in combination with antibiotics in MRSA infection treatment. Further study is also needed in an animal model to evaluate MEPL and ceftriaxone combination therapy in vivo efficacy.

## Figures and Tables

**Table 1:** Antimicrobial activity of MEPL against MRSA.

Concentration of Ceftriaxone (µg/mL)	Diameter of Zone of Inhibition (mm)	Concentration of MEPL (mg/mL)	Diameter of Zone of Inhibition (mm)
8	0	1	21 ± 2
16	0	2	24 ± 1
32	0	3	26 ± 2
64	0	4	28 ± 2
		5	29 ± 2
		6	31 ± 1
		7	32 ± 1
		8	34 ± 1

**Table 2:** Synergistic effect of MEPL and ceftriaxone sodium was determined by the checkerboard test.

MEPL (μg/mL)										
		16,000	8000	4000	2000	1000	500	250	125	62.5
Ceftriaxone sodium (μg/mL)	8000	No growth	No growth	No growth	No growth	No growth	Mild growth	Heavy Growth	Heavy Growth	Heavy Growth
	4000	No growth	No growth	No growth	No growth	Mild growth	Mild growth	Heavy Growth	Heavy Growth	Heavy Growth
	2000	No growth	No growth	No growth	No growth	Mild growth	Heavy Growth	Heavy Growth	Heavy Growth	Heavy Growth
	1000	No growth	No growth	No growth	No growth	Mild growth	Heavy Growth	Heavy Growth	Heavy Growth	Heavy Growth
	500	No growth	Mild Growth	Mild Growth	Heavy Growth	Heavy Growth	Heavy Growth	Heavy Growth	Heavy Growth	Heavy Growth
	250	No growth	Mild Growth	Heavy Growth	Heavy Growth	Heavy Growth	Heavy Growth	Heavy Growth	Heavy Growth	Heavy Growth
	125	Mild Growth	Heavy Growth	Heavy Growth	Heavy Growth	Heavy Growth	Heavy Growth	Heavy Growth	Heavy Growth	Heavy Growth
	62.5	Heavy Growth	Heavy Growth	Heavy Growth	Heavy Growth	Heavy Growth	Heavy Growth	Heavy Growth	Heavy Growth	Heavy Growth

**Table 4:** Diameter of inhibition zone interpretative criteria for *S. aureus*.

Diameter of Zone of Inhibition (mm)				
	Potency	Resistant	Intermediate	Sensitive
Ceftriaxone	30 µg	≤13	14–20	≥21

**Table 5:** PCR primer sequences for the detection of MRSA.

Primers	Oligonucleotide Primer Sequences (5′ to 3′)	Amplicon Size (bp)
*mecA* 761R	CTT GTA CCC AAT TTT GAT CCA TTT G	313
*mecA* 449F	AAA CTA CGG TAA CAT TGA TCG CAA	
16S rRNA 914R	AAC CTT GCG GTC GTA CTC CC	528
16S rRNA 387F	CGA AAG CCT GAC GGA GCA AC	

## Data Availability

Not applicable.

## Supplementary Materials

The following supporting information can be downloaded at: https://www.mdpi.com/article/10.3390/antibiotics12030477/s1. Table S1. Extracted MRSA genomic DNA concentration and purity. Table S2. The relative intensity of PCR products of the *mecA* gene for PCR optimization was obtained using ImageJ quantification software. Table S3: The relative intensity of PCR products of the *mecA* gene and 16S rRNA was obtained using ImageJ quantification software.
