# Supplementary material for: Synergistic Antimicrobial Activity of Ceftriaxone and Polyalthia longifolia Methanol (MEPL) Leaf Extract against Methicillin-Resistant Staphylococcus aureus and Modulation of mecA Gene Presence
Source: Antibiotics (Basel). 2023 Feb 27;12(3):477. doi: 10.3390/antibiotics12030477 (PMC10044657; doi:10.3390/antibiotics12030477)
Supplement: Supplementary file 1 [file antibiotics-12-00477-s001.zip › antibiotics-2182764-supplementary.pdf]

Supplementary materials

Table S1. Extracted MRSA genomic DNA concentration and purity.

No	Concentration(ng/µL)	A_{260}/A_{280}
1	25.4	1.5
2	27.1	1.2
3	30.0	0.8
4	26.5	1.1
5	29.3	1.4
6	31.5	0.9
7	28.7	1.5
8	28.2	1.4
9	27.8	1.2
10	23.6	1.5
11	29.1	0.8
12	32.5	1.1
13	35.3	1.3
14	31.9	1.2
15	30.7	1.6
16	32.4	1.4
17	34.1	1.5
18	35.0	1.6
19	36.2	1.3
20	33.5	1.7

Table S2. The relative intensity of PCR products of *the mecA* gene for PCR optimization was obtained using ImageJ quantification software.

Lane	Relative Intensity of DNA bands
1	0
2	133.778
3	676.335
4	107.778

Lane 1 = 55.0°C, Lane 2 = 56.6°C, Lane 3 = 60.0°C and Lane 4 = 61.0°C.

Table S3. The relative intensity of PCR products of *the mecA* gene and 16S rRNA was obtained using ImageJ quantification software.

Lane	Relative intensity of 16S rRNA	Relative intensity of mecA gene
1	10886.23	1667.23
2	13803.18	0
3	11675.05	4442.98
4	0	0
5	3513.28	0

Lane 1 = Treated MRSA isolate (in combination of 1000 µg/mL MEPL and 1000 µg/mL ceftriaxone), Lane 2 = Treated MRSA isolate (in combination of 2000 µg/mL MEPL and 1000 µg/mL ceftriaxone),

Lane 3 = untreated MRSA isolates, Lane 4 = blank, Lane 5 = MSSA isolate (control) Quantified value of PCR products with *mecA* gene and 16S rRNA bands.
